# A protective measles virus-derived vaccine inducing long-lasting immune responses against influenza A virus H7N9

**DOI:** 10.1038/s41541-023-00643-9

**Published:** 2023-03-24

**Authors:** Cindy Hörner, Anna H. Fiedler, Bianca S. Bodmer, Lisa Walz, Vivian A. Scheuplein, Stefan Hutzler, Mikhail N. Matrosovich, Veronika von Messling, Michael D. Mühlebach

**Affiliations:** 1 Section 4/3: Product Testing of IVMPs, Paul-Ehrlich-Institut, Paul-Ehrlich-Straße 51-59, 63225 Langen, Germany; 2 German Center for Infection Research, Gießen-Marburg-Langen, Germany; 3 Section 4/0: Research in Veterinary Medicine, Paul-Ehrlich-Institut, Paul-Ehrlich-Straße 51-59, 63225 Langen, Germany; 4 Institute of Virology, Philipps University, Marburg, Germany; 5 Present Address: Institute of Molecular Virology and Cell Biology, Friedrich-Loeffler-Institut, 17493 Greifswald-Insel Riems, Germany

**Keywords:** Influenza virus, Live attenuated vaccines, Live attenuated vaccines, Viral infection, Live attenuated vaccines

## Abstract

A novel Influenza A virus (subtype H7N9) emerged in spring 2013 and caused considerable mortality in zoonotically infected patients. To be prepared for potential pandemics, broadly effective and safe vaccines are crucial. Recombinant measles virus (MeV) encoding antigens of foreign pathogens constitutes a promising vector platform to generate novel vaccines. To characterize the efficacy of H7N9 antigens in a prototypic vaccine platform technology, we generated MeVs encoding either neuraminidase (N9) or hemagglutinin (H7). Moraten vaccine strain-derived vaccine candidates were rescued; they replicated with efficiency comparable to that of the measles vaccine, robustly expressed H7 and N9, and were genetically stable over 10 passages. Immunization of MeV-susceptible mice triggered the production of antibodies against H7 and N9, including hemagglutination-inhibiting and neutralizing antibodies induced by MV_vac2_-H7(P) and neuraminidase-inhibiting antibodies by MV_vac2_-N9(P). Vaccinated mice also developed long-lasting H7- and N9-specific T cells. Both MV_vac2_-H7(P) and MV_vac2_-N9(P)-vaccinated mice were protected from lethal H7N9 challenge.

## Introduction

Vaccines against influenza are in use since the end of the 1930s^1^. Different approaches using inactivated trivalent vaccines (TIV), including inactivated whole virus, split and subunit vaccines, or live attenuated influenza vaccines (LAIV) have already been developed and authorized^2^. Due to the high mutation rate of influenza viruses and reassortment of genomic segments yielding new, altered seasonal or even novel pandemic influenza strains, new vaccines have to be produced annually^3^. The production of these vaccines typically rely on the amplification of live influenza virus in embryonated chicken eggs^3,4^, which have limited production capacity^4^. These limits have been responsible for vaccine shortages under special circumstances, such as the pandemics declared in June 2009 for H1N1^5^, when the delayed availability of vaccines highlighted that classical influenza vaccine production strategies may not be fast enough to prevent the pandemic spread of a novel influenza virus^3^. A solution for this issue may be found in efficient and fast vaccine platform technologies with the opportunity to easily exchange the critical antigens of newly emerged or genetically drifted influenza strains^3,4^.

A novel H7N9 avian influenza A virus (IAV) lineage may be a challenge of this kind. Since its emergence in 2013^6^, 39% of more than 1500 H7N9-infected patients have succumbed to zoonotic infection with a few human-to-human transmissions^7^. Although the infectivity of this virus for humans is still quite low, genetic changes facilitating an adaption to mammalian receptors^8^ and an efficient spread between ferrets by contact^9,10^ reveal an adaption to mammalian hosts^11,12^ and indicate that an enhanced human-to-human transmission is possible by genetic drift of the virus. This potential for pandemic mandates the development of viable alternative technologies in case of rapid, world-wide spread as observed during the recent COVID-19 pandemic. First and still most authorized COVID-19 vaccines of the Western World rely on vaccine platform technologies (i.e. mRNA vaccines Comirnaty and Spikevax, AdV-derived vaccines Vaxzevria and Jcovden vs. protein vaccines Nuvaxovid and VidPrevtyn Beta, inactivated virus COVID19 vaccine Valneva^13,14^) that allow rapid adaption to new challenges. However, to generate a successful vaccine on the basis of a platform technology, understanding of the protective efficacy of selected antigens against variants of pathogen subtypes is crucial.

Among promising vaccine platform candidates are vaccine strain-derived, replicating recombinant measles virus (MeV) vaccines. MeV vaccine strains are strongly immunogenic and reveal an excellent safety record evidenced with billions of doses being used for measles immunization since 1963^15,16^. A single immunization results in strong humoral as well as cellular immune responses in 85% of vaccinees, which probably provides life-long protection^15,17^. To reach the high level of coverage for protective herd-immunity, a two-dose immunization schedule is recommended, nevertheless^18^. Moreover, the manufacturing of measles vaccines is well established^19^ on industrial scale. Reverse genetics systems for recombinant MeV have been generated, which allow the insertion of extra gene segments of up to 6 kb into recombinant MeV^19,20^. Thereby, a wide range of MeV-derived vaccines targeting e.g. HBV^21^, HIV^22^, Chikungunya virus (CHIKV)^23^, Middle-East Respiratory Syndrome Coronavirus (MERS-CoV)^24^, Zika virus (ZIKV)^25^ or lately SARS-CoV-2^26,27^ have been generated by expressing respective antigens mediating protection. Immunization of MeV-susceptible receptor-transgenic, type I interferon receptor-deficient IFNAR^−/−^-CD46Ge mice^28^ or non-human primates^22^ induced significant immune responses against the inserted antigens^21–24^ even in animals with pre-formed anti-measles immunity^22,23^. These immune responses were shown to be protective against e.g. CHIKV^23^, MERS-CoV^24^ and ZIKV^25^. Furthermore, MV-CHIKV has successfully completed phase I and phase II clinical trials, where it efficiently induced immune responses against CHIKV irrespective of pre-existing α-MeV antibodies^29,30^. Therefore, recombinant MeV can be regarded as one of the promising vector platform technologies besides e.g. mRNA or adenoviral vector-derived systems to generate pandemic vaccines against emerging infections. Therefore, MeV-derived vaccine candidates can inherently be utilized to qualify critical parameters of respective vaccine vectors such as choice and format of foreign antigens to become expressed in the pre-pandemic setting.

This study aimed to characterize pre-pandemic MeV-derived model vaccines against H7N9 and to compare the protective efficacy of immune responses against hemagglutinin (H7) and neuraminidase (N9) induced by these vaccines. To analyze the MeV-derived vaccines for protection against influenza infections, we generated MeVs expressing the full-length H7 or N9 of IAV subtype H7N9. These antigens are the main components of traditional split vaccines. Antibodies directed against these glycoproteins can inhibit their critical biological functions, i.e. receptor-binding and membrane fusion or enzymatic cleavage of sialic acids, and thus prevent viral replication^31,32^ and protect from viral pathology^8,33–36^. For this purpose, we cloned H7- or N9-encoding ORFs of A/Shanghai/2/2013 (H7N9) virus into the respective additional transcription units (ATU) following the P (post P) or the H gene cassette (post H) of the MeV Moraten vaccine strain genome (MV_vac2_). Both post P (MV_vac2-_H7(P); MV_vac2_-N9(P)) or post H (MV_vac2-_H7(H); MV_vac2_-N9(H)) positions were tested to take advantage of the transcriptional gradient in MeV genomes. The vaccine candidates were rescued and expressed the inserted antigens without impact on the recombinant virus replication. Immunization of IFNAR^−/−^-CD46Ge mice induced high titers of antibodies and antigen-specific T cells. Antibodies induced by immunization with MV-H7(P) had hemagglutination-inhibiting as well as virus-neutralizing capacity, whereas antibodies induced by both H7- and N9-encoding vaccines inhibited neuraminidase activity when using H7N9-presenting virus particles as substrates. Moreover, cellular immune responses were still detectable more than two years after vaccination of juvenile mice at the end of their natural life-span. Finally, immunization with either H7 or N9 encoding candidate vaccines protected IFNAR^−/−^-CD46Ge mice from lethal H7N9 challenge. Our data thereby demonstrate on the basis of MeV as an efficient vaccine platform that both H7 and N9 are valuable antigen structures also in this technology to target potentially pandemic, highly pathogenic influenza virus subtypes.

## Results

### Generation of recombinant vaccine-strain MeV expressing H7 or N9 of IAV H7N9

**Fig. 1:**
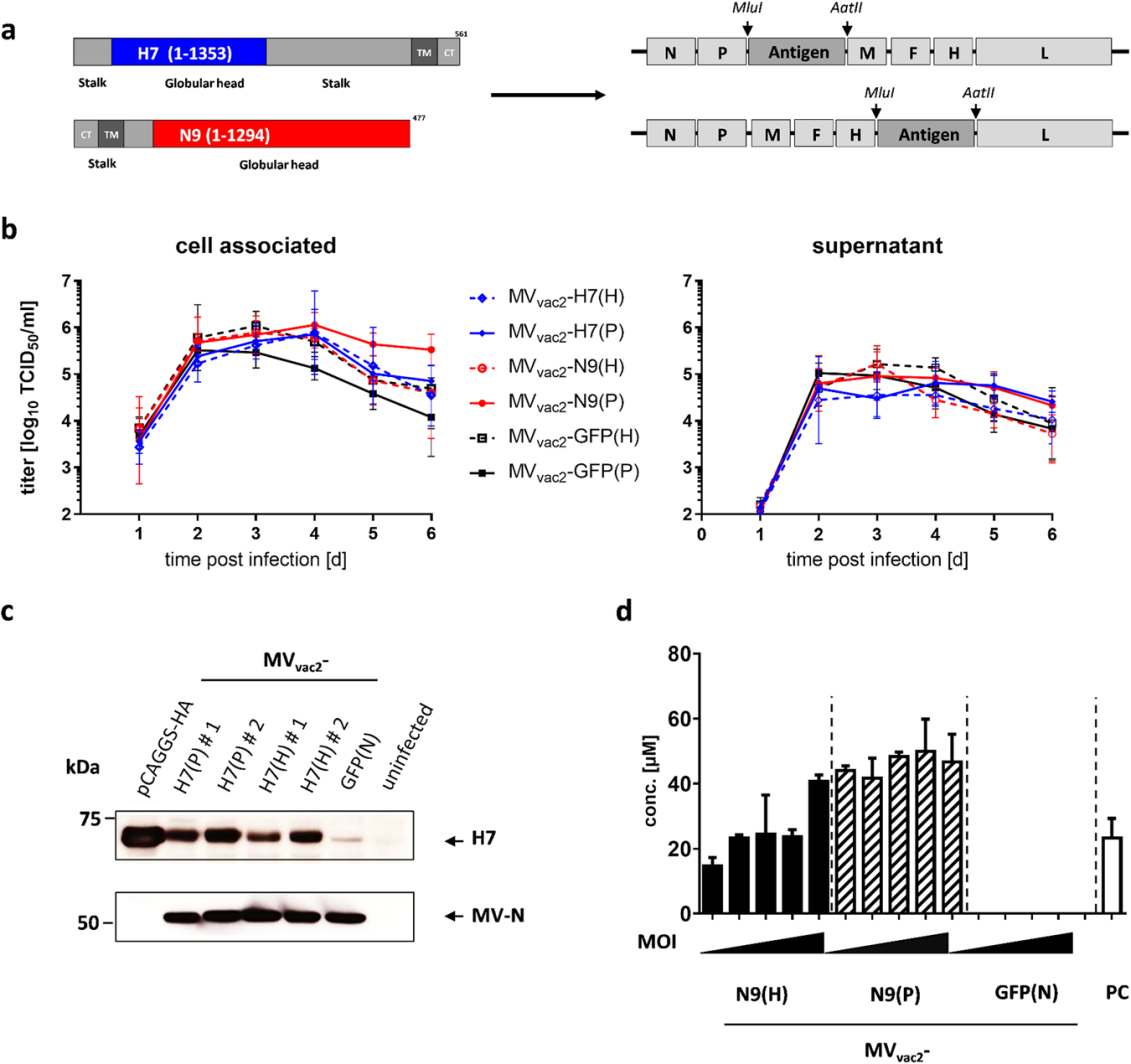
Generation and characterization of MV_vac2_-H7 and MV_vac2_-N9. **a** Schematic depiction of full-length hemagglutinin H7 and neuraminidase N9 antigens (left scheme) expressed by recombinant MV_vac2_ genomes (right scheme). Antigens or antigen-encoding genes are depicted in dark grey with globular heads coloured (H7, blue; N9, red); MeV viral gene cassettes (in light grey) are annotated. *MluI* and *AatII* restriction sites used for cloning of antigen-genes into post P or post H ATU are highlighted. **b** Growth kinetics of recombinant MeV on Vero cells infected at an MOI of 0.03 with MV_vac2_-H7(H), MV_vac2_-H7(P), MV_vac2_-N9(H), MV_vac2_-N9(P), or MV_vac2_-GFP. Samples collected at the indicated time points post infection were titrated on Vero cells. Geometric means of three independent experiments are presented, error bars indicate geometric SD. **c** Immunoblot analysis of Vero cells infected at an MOI of 0.03 with two independent virus clones of MV_vac2_-H7(H) and MV_vac2_-H7(P) or MV_vac2_-GFP(N) as depicted. Uninfected cells served as mock, pGAGGS-H7 as positive control. Blots were probed using rabbit serum reactive against H7N1 (upper blot) or, after stripping of the membrane, mAb reactive against MV-N (lower blot) and thus were derived from the same experiment. Arrows indicate specific bands. **d** Neuraminidase activity assay for verification of N9 expression using MUNANA as substrate. Uninfected and MV_vac2_-GFP(N)-infected cells served as negative and pGAGGS-N9 transfected cells as positive control (PC); *n* = 3. Error bars indicate SEM.

Since the envelope glycoproteins hemagglutinin and neuraminidase are major protective antigens of IAV^34,36^, both antigens were chosen to be expressed by recombinant MV_vac2_ to analyze measles virus platform-based vaccine candidates against IAV H7N9. For this purpose, full-length ORFs encoding hemagglutinin subtype H7 and neuraminidase subtype N9 were each cloned into two different additional transcription units (ATUs) either after the P (post P) or after the H (post H) gene cassettes of MV_vac2_ (Fig. 1a). This strategy takes advantage of the MeV transcription gradient^37^ to generate recombinant MeV which are high or dampened in the transcription of foreign antigens´ mRNA. Clones of all recombinant viruses, i.e. MV_vac2_-H7(P), MV_vac2_-H7(H), MV_vac2_-N9(P), and MV_vac2_-N9(H), were rescued and passaged until passage 10 (P10) with titers up to 1.2 × 10^8^ TCID_50_/ml for MV_vac2_-H7(P), 1.9 × 10^8^ TCID_50_/ml for MV_vac2_-H7(H), 6.9 × 10^7^ TCID_50_/ml for MV_vac2_-N9(P) and 5.9 × 10^7^ TCID_50_/ml for MV_vac2_-N9(H). The genomic integrity of the additional transgene expression cassettes was demonstrated after short- (P3) (Supplementary Fig. 1) or long-term passage (P10) by sequencing of respective regions in the vaccine genomes. The insertion of the extra genetic elements did not significantly affect viral growth in Vero cells compared to control viruses expressing GFP in the respective ATUs (Fig. 1b). The expression of H7 (Fig. 1c, Supplementary Fig. 2) was demonstrated by Western Blot analysis, while the enzymatic activity of the expressed N9 (Fig. 1d) was demonstrated in vaccine virus-infected cells using the neuraminidase substrate MUNANA. As expected, both antigens were expressed about two-fold more efficiently after insertion into the post P than into the post H ATU. Thus, the generation of MV_vac2_-derived vaccines expressing the hemagglutinin or neuraminidase of IAV H7N9 was achieved yielding genetically stable, high-titer vaccines without replication deficiencies and with significant expression of the additional antigens. This indicates a good compatibility of the chosen antigens with vaccine technologies derived from viral vector platforms.

### MV_vac2_-H7(P) and MV_vac2_-N9(P) induce significant humoral immunity in immunized MeV-susceptible mice

**Fig. 2:**
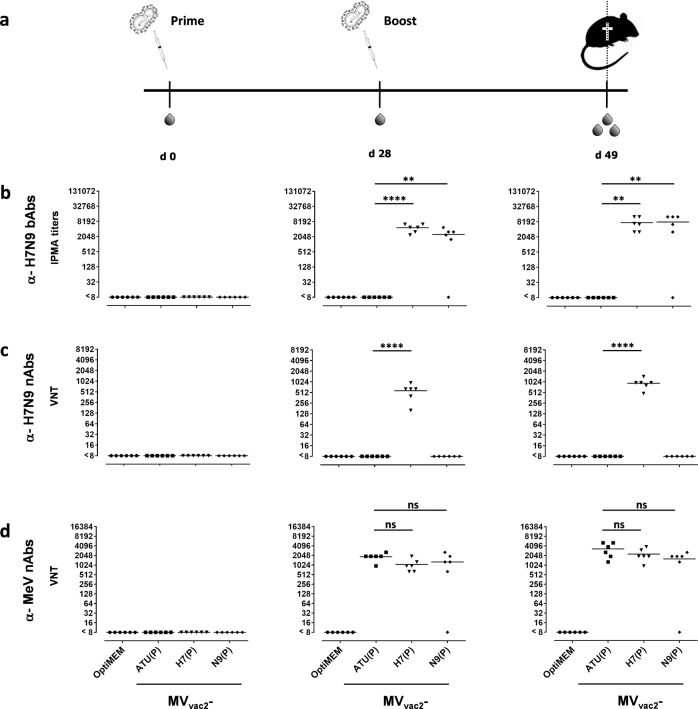
Induction of H7N9-specific binding and neutralizing antibodies. **a** Blood of mice vaccinated on days 0 and 28 with indicated viruses was sampled on day 0, 21 and 49. Sera were analyzed for (**b**) α-H7N9 binding antibodies (bAbs) and (**c**) H7N9 neutralizing antibodies (nAbs) as well as (**d**) MeV nAbs. Medium (OptiMEM) or empty measles vaccine (ATU(P)) inoculated mice served as controls. **b** Total α-H7N9 bAbs were determined as the reciprocal of the highest serum dilution staining H7N9-A/PR/8/34 infected cells in IPMA. **c**, **d** Virus neutralizing titers (VNT) were calculated as the reciprocal of the highest serum dilution completely neutralizing virus infectivity. Dots represent single animals (*n* = 6); horizontal line represents mean per group. Y-axis starts at detection limit; all mice at detection limit had no detectable VNT. ns, not significant; **, *p* < 0.01; ****, *p* < 0.0001 (non-parametric One-way ANOVA).

MV_vac2_-H7(P) and MV_vac2_-N9(P) were chosen for immunization experiments due to the stronger expression of the inserted antigens (Fig. 1c, d). For vaccination, MeV-susceptible transgenic IFNAR^−/−^-CD46GE mice were vaccinated with MV_vac2_-H7(P), MV_vac2_-N9(P), medium (OptiMEM) or measles control vaccine (MV_vac2_-ATU(P)) in a prime-boost set-up using a dose of 1×10^5^ TCID_50_ of recombinant vaccine virus for each vaccination (Fig. 2a). First, sera collected before (d 0) as well as after the first (d 28) and second immunization (d 49) were analyzed for total H7- or N9-binding antibodies (bAbs) via immune peroxidase monolayer assay (IPMA) (Fig. 2b). For this purpose, MDCK cells infected with recombinant H7N9 A/Shanghai/2/2013-A/PR/8/34, thereby expressing all influenza virus antigens including H7 and N9, were used as targets. While vaccination with MV_vac2_-H7(P) or MV_vac2_-N9(P) induced bAbs with mean titers of 4667 ± 1633 or 2535 ± 1631 after prime immunization, respectively, sera of medium or vector control mice did not include antibodies above IPMA detection level. A single mouse vaccinated with MV_vac2_-N9(P) did not react with any antibodies targeting IVA or MeV, most likely reflecting a general failure of vaccination of this individual animal. Thus, MV_vac2_-H7(P) induced 1.8-fold higher bAb titers than MV_vac2_-N9(P) after the prime immunization, but a homologous booster increased bAbs in both cohorts to a comparable titer around 8000.

Next, we analyzed the virus-neutralizing titers (VNTs) of these antibodies against the recombinant H7N9 A/Shanghai/2/2013-A/PR/8/34, which expresses the H7 and N9 in the backbone of PR8 IAV strain (Fig. 2c). Only the antibodies induced by MV_vac2_-H7(P), but not those by MV_vac2_-N9(P) revealed neutralizing capacity. The neutralizing capacity of these Abs were boosted 1.6-fold by the second immunization (from 573 ± 270 to 933 ± 311 VNT). As expected, no nAbs neutralizing recombinant H7N9-PR8 virus were detectable in sera of mock or vector control mice. To control effective immunization, neutralization of MeV by the sera of the immunized mice was quantified, as well (Fig. 2d). As expected, antibodies neutralizing MeV were detectable in all immunized but not in mock control mice or in mice before immunization. High anti-MeV VNTs of 1,867 ± 513 (MV_vac2_-ATU(P)), 1,067 ± 482 (MV_vac2_-H7(P)), and 1,281 ± 902 (MV_vac2_-N9(P)) after the first and 3,307 ± 1640 (MV_vac2_-ATU(P)), 2,267 ± 1014 (MV_vac2_-H7(P)), and 1,601 ± 879 (MV_vac2_-N9(P)) after the second immunization were found, revealing robust induction and boosting of measles immunity in all vaccine groups without significant differences. Interestingly, the single mouse immunized with MV_vac2_-N9(P) that had no detectable α-influenza nAbs did also not produce any α-MeV nAbs indicating a general failure of vaccination in this animal.

The results demonstrate that both MV_vac2_-H7(P) and MV_vac2_-N9(P) are potent inducers of humoral immune responses directed against H7 and N9, respectively. However, only MV_vac2_-H7(P), but not MV_vac2_-N9(P) induces virus-neutralizing Abs.

### Antibodies induced by MV_vac2_-based H7N9 vaccines inhibit biological activities of influenza H7N9 glycoproteins

**Fig. 3:**
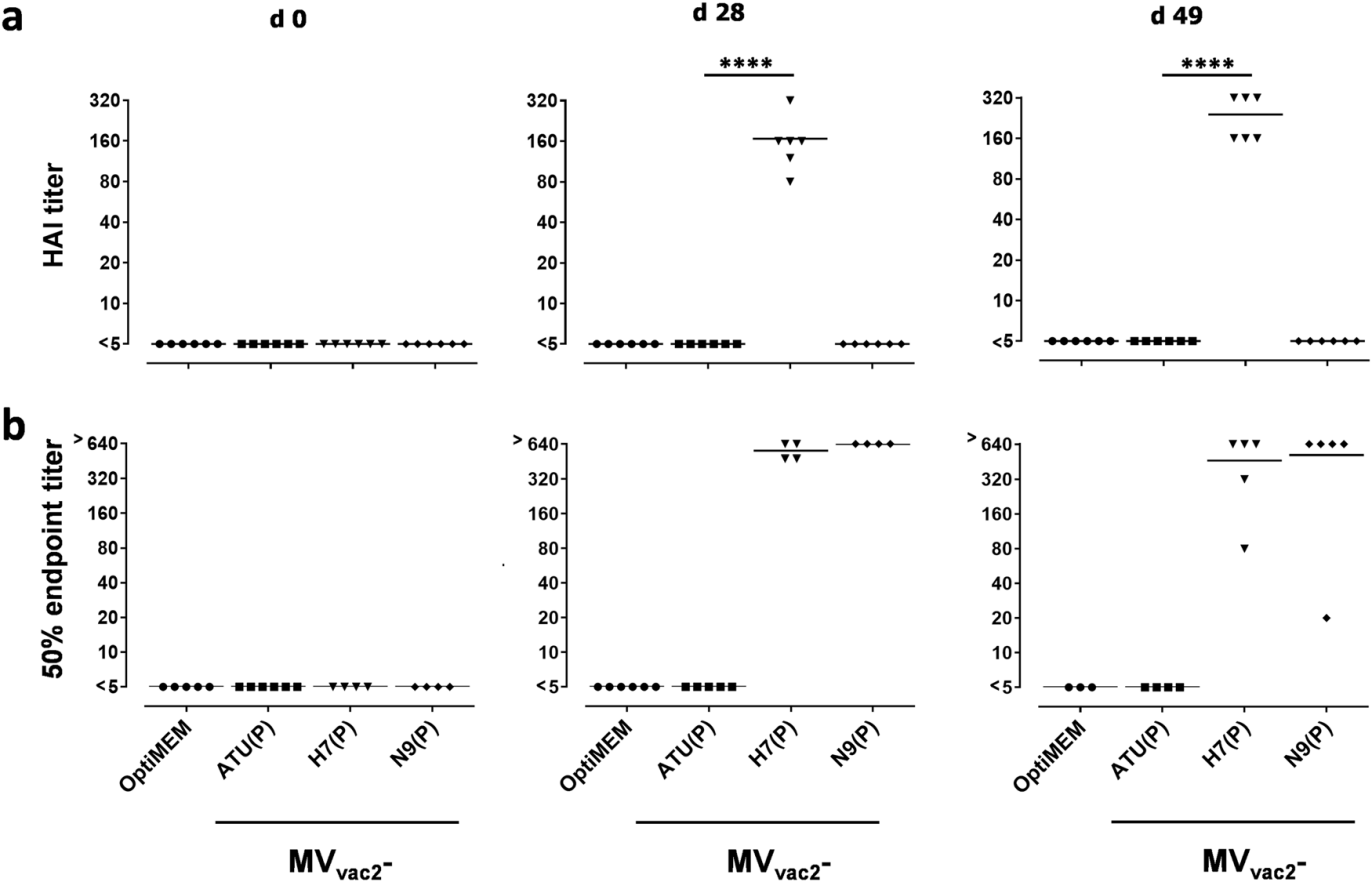
Inhibition of hemagglutination and neuraminidase activity by sera of vaccinated mice. Hemagglutination and neuraminidase inhibition activity of serum antibodies were assayed on days 0, 28, and 49 after immunization as indicated in Fig. 2a. **a** Hemagglutination inhibiting titers (HAI titers) were calculated as the highest serum dilution abolishing the hemagglutination activity of H7N9-A/PR/8/34. **b** Neuraminidase inhibition was calculated as the 50% NA inhibitory concentration (IC_50_) after incubation of H7N9-A/PR/8/34 with mice sera in enzyme-linked lectin assay (ELLA). Dots represent single animals (*n* = 6); horizontal line represents mean per group. Y-axis starts at detection limit; all mice at detection limit had no detectable titers. ****, *p* < 0.0001 (non-parametric One-way ANOVA).

To assess the other modes of action besides neutralization of the antibodies which are induced by MV_vac2_-H7(P) or MV_vac2_-N9(P) on influenza H7N9 infectivity and spread, we determined hemagglutination as well as neuraminidase inhibition titers (Fig. 3). As expected, sera of mice immunized with MV_vac2_-H7(P), but not of mock-treated animals or those vaccinated using MV_vac2_-ATU(P) or MV_vac2_-N9(P) inhibited hemagglutination of chicken erythrocytes (hemagglutination inhibition, HAI) by H7 incorporated in the envelope of H7N9-PR8 virus particles (Fig. 3a). The HAI titer of 167 ± 82 after the first immunization was 1.4-fold boosted to 240 ± 88 after the second immunization. To analyze whether neuraminidase-activity is inhibited by the humoral immune response, we performed an enzyme-linked lectin assay (ELLA)^38^. Here, NA-inhibition titers in 5 out of 6 mice vaccinated with MV_vac2_-N9(P), and rather unexpectedly in 3 out of 5 mice vaccinated with MV_vac2_-H7(P) were above 640 (50% endpoint titer) when testing N9 incorporated into H7N9-PR8 particles (Fig. 3b).

These data indicate that MV_vac2_-H7(P)- or MV_vac2_-N9(P)-induced antibodies are not only able to bind to H7N9 glycoproteins, but are also able to inhibit their receptor-binding and enzymatic activities and thus influenza virus pathogenicity.

### Antigen-specific cellular anti-influenza immune responses in animals vaccinated with MeV-derived vaccines

**Fig. 4:**
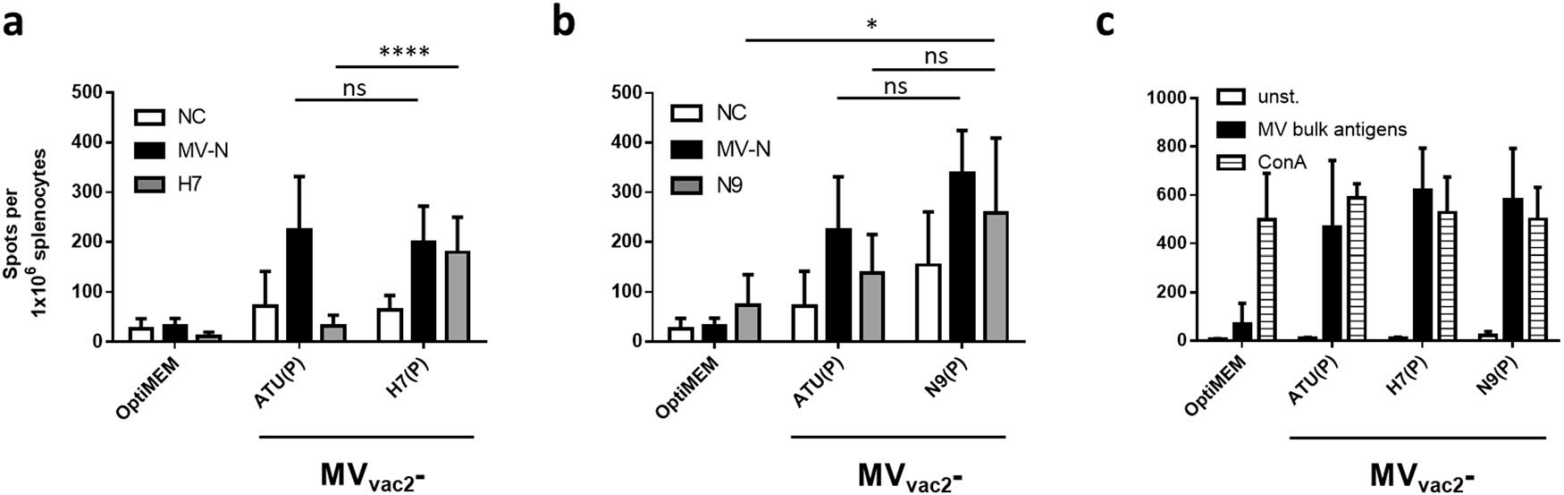
Secretion of IFN-γ after antigen-specific re-stimulation of splenocytes. IFN-γ ELISpot analysis of murine splenocytes isolated 4 d after boost immunization. **a** H7- or (**b**) N9-specific T cells were detected after co-culture of splenocytes with JAWSII dendritic cell lines transgenic for MeV-N (black columns), H7, or N9 (grey columns). **a, b** Untransduced cells (NC) or (**c**) medium (unst.) served as negative controls (white columns). **c** Splenocytes were stimulated with 10 μg/ml MeV bulk antigens (black columns) or ConA (striped columns) as positive controls. Presented are means per group (*n* = 6), error bars indicate SD. ns, not significant; *, *p* < 0.05; ****, *p* < 0.0001 (non-parametric One-way ANOVA).

Since a strong correlation between T cell responses and influenza clearance has been observed in various human trials^39–41^ and in a mouse model^42^, we analyzed the ability of MV_vac2_-H7(P) or MV_vac2_-N9(P) to induce cellular immunity against H7N9. The reactivity of splenocytes of the vaccinated mice was determined by IFN-γ ELISpot after re-stimulation with H7- or N9-presenting syngenic dendritic cell (DC) clones four days after the booster immunization. For this purpose, transgenic DC clones based on the syngenic cell lines JAWSII and DC2.4 were generated by lentiviral transduction with H7- or N9-encoding gene transfer vectors. The activation of splenocytes was measured by IFN-γ ELIspot assay after co-culture of splenocytes with these DC clones or a clone stably expressing MeV-N^24^ (Fig. 4a, b, Supplementary Fig 3). In addition, splenocytes of all groups were stimulated with the antigen-independent polyclonal stimulator Concanavalin A (ConA) to test the general reactivity of the samples and with MeV-bulk antigens to control anti-MeV reactivity (in addition to the MeV N-expressing DC clone). All mice showed similar numbers of IFN-γ secreting cells upon ConA treatment indicating that splenocytes of all animals were reactive (Fig. 4c). Additionally, no significant difference in IFN-γ secreting cells could be observed after re-stimulation with JAWSII-MV-N (Fig. 4a, b) or MeV bulk antigen (Fig. 4c) between splenocytes of mice vaccinated with MV_vac2_-ATU(P), MV_vac2_-H7(P), or MV_vac2_-N9(P). Thus, all mice were similarly susceptible to MeV vaccination. However, a significant 5.7-fold increase (179 ± 70 vs. 32 ± 22) in the number of IFN-γ secreting splenocytes from MV_vac2_-H7(P)-immunized mice compared to those vaccinated by the vector control MV_vac2_-ATU(P) was observed upon H7-specific re-stimulation (Fig. 4a). This observation indicates that only MV_vac2_-H7(P) induced influenza-specific T cells while both vaccine and vector control triggered similarly α-MeV T cells. The latter holds also true for the MV_vac2_-N9(P) vaccine. However, although the number of IFN-γ secreting cells among splenocytes of MV_vac2_-N9(P)-vaccinated mice was 1.9-fold higher in comparison to those of MV_vac2_-ATU(P) vaccinated mice after re-stimulation with N9, this difference was not statistically significant due to large variation within both groups (Fig. 4b). Interestingly, splenocytes of MV_vac2_-N9(P) vaccinated mice revealed high background activation upon contact with JAWSII cells irrespective of the presented antigen. Replication of these experiments using transgenic DC2.4 cell clones for antigen presentation gave similar results (Supplementary Fig. 3) thus excluding JAWSII-specific experimental artifacts.

In conclusion, we could demonstrate significant antigen-specific cellular immune responses against H7 in MV_vac2_-H7(P) vaccinated mice. While our data also indicate cellular immune responses against N9 in respective vaccinated mice, these results lack statistical significance due to comparatively high background reactivity or background stimulation and heterogeneity of the observed reactivity. Thus, both arms of the adaptive immune system are induced by H7- or N9-expressing measles vaccine vectors.

### Long-term cellular immunity in individual mice after vaccination with MV_vac2_-H7(P)

**Fig. 5:**
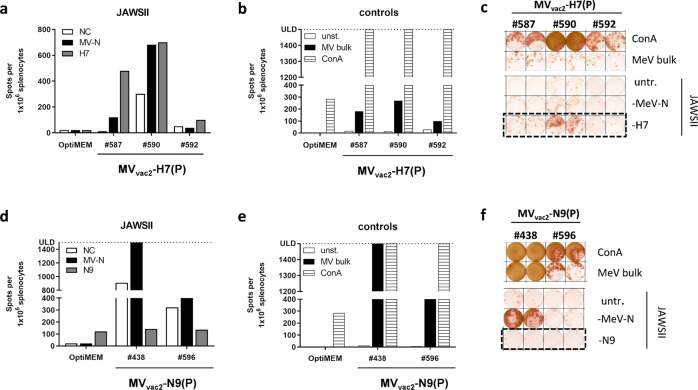
Long-term H7N9-specific cellular immunity of aged mice. **a**–**c** IFN-γ ELISpot assay after antigen-specific re-stimulation of murine splenocytes isolated 2 years after boost immunization with MV_vac2_-H7(P) (**a–c**) or with MV_vac2_-N9(P) (**d**–**f**). Untransduced cells (**a**, **d**) or medium (**b**, **e**) served as negative controls (white columns). H7- or N9-specific T cells were detected after co-culture of splenocytes with JAWSII dendritic cell lines transgenic for MeV-N (black columns), H7, or N9 (grey colums). Untransduced cells (NC) (**a**, **d**) or medium (unst.) (**b**, **e**) served as negative controls (white columns) while stimulation with 10 μg/ml MV bulk antigens (black columns) or ConA (striped columns) (**b**, **e**) served as positive controls. Results of individual mice are shown.

For one cohort of mice, analysis followed the prime-boost vaccination after over two years of interim time. Three mice vaccinated with MV_vac2_-H7(P) and two mice vaccinated with MV_vac2_-N9(P) remained available for the analysis, which were used to determine the longevity of cellular responses by performing IFN-γ ELISpot with their splenocytes as described above. Interestingly, we could detect clear H7-specific responses in two of three analyzed animals (#590, #587) and enhanced reactivity also in the third mouse #592 (Fig. 5a, c). For animals #590 and #587, the number of reactive T cells was even in a similar range compared to the experiments determining cellular responses 4 days post boost vaccination. A single mock control animal from the same experiment did neither show H7- (Fig. 5a) nor MeV-specific responses (Fig. 5a, b), but was still responsive to ConA treatment (Fig. 5b) the latter indicating the preserved reactivity of the splenocytes, in principle. However, the two N9-vaccinated mice did not show N9-specific re-stimulation over the background level (Fig. 5d–f).

Albeit quite limited in numbers of tested individual animals (due to considerable loss of animals over the long time course of the experiment), these data indicate that in principle geriatric mice may still profit from immunity when vaccinated with MeV-platform-derived vaccines in their youth.

### Protection of H7 and N9-vaccinated mice against lethal influenza virus challenge

**Fig. 6:**
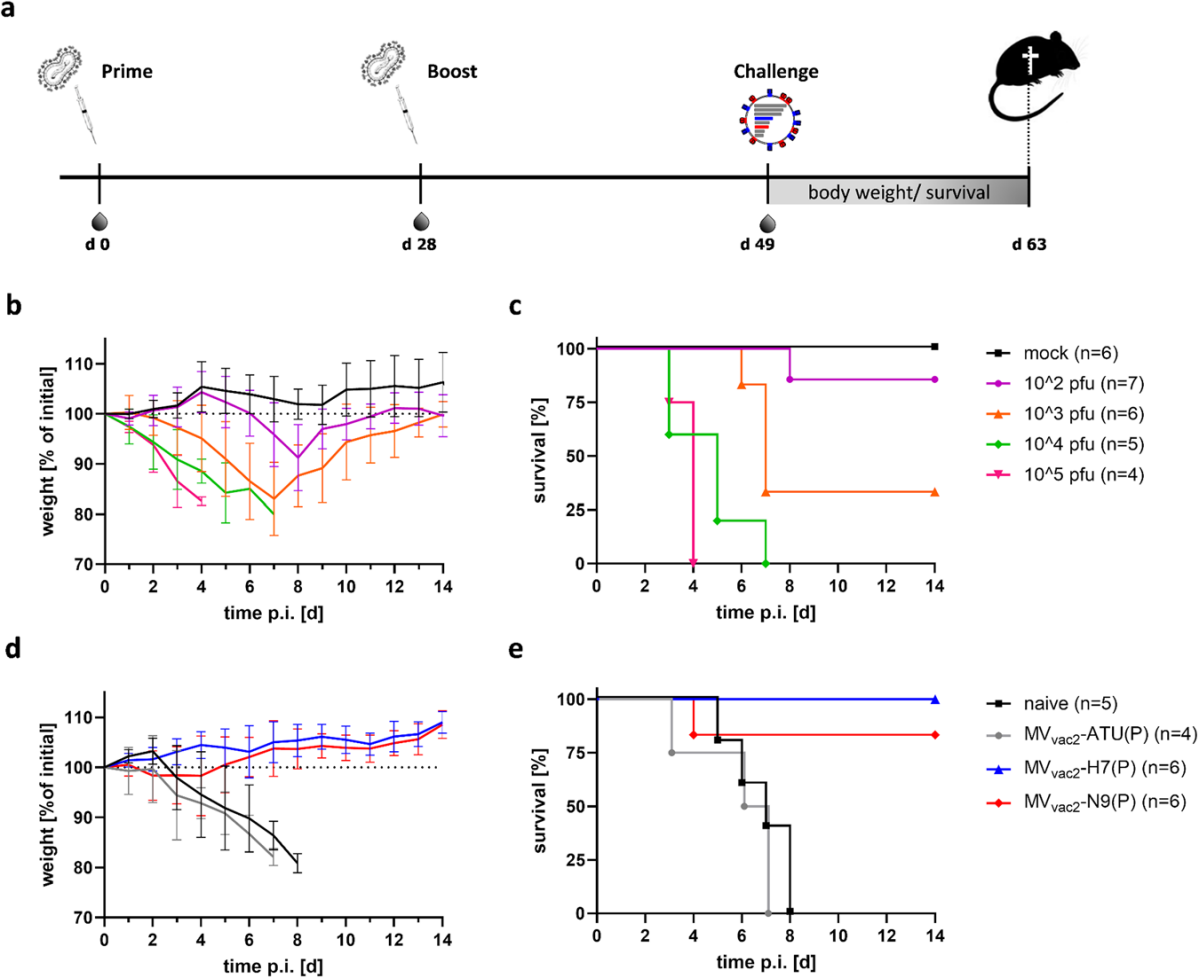
H7N9 challenge experiments. **a** Vaccination and challenge scheme used in challenge experiments shown in (**d**, **e**). **b, c** In vivo titration of the 50% lethal dose (LD_50_) of intranasal H7N9 challenge virus used in IFNAR^−/−^-CD46Ge mice ranging between 10^2^-10^5^ pfu/mouse. For survival, the weight cutoff of ≥80% of initial body weight was used. **d**, **e** H7N9 challenge of vaccinated mice with a determined dose of 10× LD_50_ (1 × 10^4^ pfu). Error bars indicate SD of means.

In a final set of experiments, we wanted to test, if and which of the immune responses detected in previous analyses are protective in a challenge experiment of respectively vaccinated animals. The challenge was performed using the available wild-type isolate A/Anhui/1/2013 (H7N9) with H7 and N9 antigenically identical to those of A/Shanghai/2/2013 (H7N9) employed for the generation of the MeV-derived vaccine. To establish an appropriate challenge dose in vivo, the LD_50_ dose was first determined in the IFNAR^−/−^-CD46Ge mouse strain susceptible for vaccination with MeV-derived vaccines. For this purpose, naïve mice of this strain were infected intranasally with 10-fold ascending doses of the virus ranging between 10^2^ and 10^5^ pfu (Fig. 6b, c). All mice in the groups infected with the highest infectious doses of 10^5^ and 10^4^ pfu (Fig. 6b) had to be sacrificed 4 to 7 days postinfection due to reaching the 80% weight limit, revealing a dose-dependent effect of weight loss and killing. Both lower infection doses (10^3^ and 10^2^ pfu/animal) resulted in partial survival by 33% (for 10^3^ pfu) and 86% (for 10^2^ pfu). Weight curves (Fig. 6b) correlated well with the used infection dose and survival rates (Fig. 6c). Using a sigmoidal non-linear standard curve model for interpolation of log_10_ (dose) and death rates resulted in a calculated LD_50_ value of 2.98 log_10_ pfu for the used IFNAR^−/−^-CD46Ge strain. For the following challenge experiments of vaccinated mice, infection doses of 10x LD_50_, i.e. 10^4^ pfu/mouse, were used.

To analyze the protective capacity of the immune responses which are induced by MV_vac2_-H7(P) or MV_vac2_-N9(P), mice were immunized with either vaccine, the control vector MV_vac2_-ATU(P), or placebo (OptiMEM) as described above. Three weeks after the second immunization, vaccinated mice were challenged. While naïve control animals and the MV_vac2_-ATU(P) vector control mice had to be sacrificed due to the pathogenesis of H7N9 infection, 100% (6/6) of mice received the H7 vaccine and 83% of mice (5/6) vaccinated with the N9 vaccine were protected. H7-vaccinated mice did not experience any weight drop after challenge, while N9-vaccinated mice fully recovered after 4 days p.i. after an initial slight drop in body weight. One mouse in the N9-vaccine group developed progressive weight loss and therefore had to be sacrificed on day 4 p.i. (Fig. 6d, e). Therefore, vaccines inducing responses against either of the chosen antigens revealed remarkable protection in this uniformly lethal setting.

## Discussion

In this study, we aimed at evaluating the performance of two major influenza surface antigens, hemagglutinin and neuraminidase, to qualify their usefulness to generate vaccine platform-based vaccine candidates targeting IAV subtype H7N9 in the pre-pandemic setting. For this purpose, we took advantage of recombinant, vaccine strain-derived MeV and successfully generated two genetically stable vaccine prototypes that expressed either H7 or N9 in high amounts without losing characteristic vaccine properties. Both vaccine candidates induced functional antibody responses targeting the characteristic activities of either target protein. Also, considerable antigen-specific cellular immune responses were induced against H7 or N9. The respective immune responses resulted in complete protection of H7-vaccinated animals and protection of 90% of N9-vaccinated animals against lethal influenza infection challenge. Most remarkable was the longevity of H7-specific T cells that were still detected in mice approaching the end of their natural life-span, although they had been vaccinated as juveniles.

Such distinct longevity of immunity is well-described for MeV after natural infection that results in life-long protection^43^. A comparable trend is assumed for the measles vaccine, which is a life-attenuated version of MeV, still closely resembling the high and early immune cell tropism of the parental virus^44–48^. Parallel to the drastic reduction of circulating MeV in the population due to the WHO measles eradication campaign^49^, some progressive decrease of protection afforded by the MeV vaccine has been observed over the last two decades^50^. Although this correlation has put a slight question mark about comparability of measles immunity after infection or vaccination, the stability of measles immunity is anyway extraordinary. Therefore, our data confirm at least for the H7 antigen that also the encoded foreign antigens can profit from these vaccine platform properties of recombinant MeV. Moreover, the activation of both arms of the adaptive immune system is triggered by our live-attenuated vaccine candidates and results in the induction of both functional antibody responses and considerable antigen-specific T cells targeting H7 or N9. These results demonstrate the advantages of this technology. In contrast, adjuvanted proteins as used in the classical split vaccine or inactivated virus approaches are mainly inducing antibody, but are doing less well in boosting of T cell responses^51^.

Such classical approaches to generate influenza vaccines suffer from the high antigenic variability of influenza viruses especially of the hemagglutinin, which requires annual adaptation to forecasted subtypes and serotypes of circulating strains that can be expected to dominate the up-coming influenza season^52^. While this process is routinely established for the usual fluctuation of antigenic profiles of circulating strains, a potentially up-coming influenza pandemic will force tough decisions on the allocation of limited production resources (usually in embryonated SPF chicken eggs), as has become evident for the H1N1/2009 influenza pandemic^53^. Until truly broadly protective vaccines covering at least the antigenic profiles of specific subtypes are established, applications of vaccine platform-based technologies such as viral vectors or mRNA vaccines can be helpful to fill the gap, a principle that has been impressively demonstrated by the mRNA and AdV-vector derived COVID-19 vaccines during the recent SARS-CoV-2 pandemic. Therefore, the validation of both H7 and N9 as designated cargo antigens of platform-based vaccines will help to design such future vaccines. For neuraminidase-directed responses, even a broader reactivity can be expected given the induction of antibodies against conserved epitopes of this protein^54^. For neuraminidase-targeted antibodies triggered during infection it has been anecdotally shown for N9, that monoclonal antibodies derived from such patients may be even cross-reactive across subtypes^55^ and animal models have indeed shown protection against the heterologous challenge from the same NA subtype^56^.

Nevertheless, the immunological properties of the chosen antigens seem also to influence the immune responses, at least with respect to longevity of the latter. In contrast to the H7-vaccine, no N9-directed T-cell responses could be identified in the geriatric mouse cohort. This finding could be due to the anecdotal character of this report comprising just two animals in the latter cohort due to most animals had reached their maximal lifespan and had to be sacrificed before T-cell responses could be measured. However, sustainability of antibody responses after vaccination with adjuvanted protein has already been described for H7 in human vaccine trials^57^ and our data are thus consistent with these observations. Moreover, the activity of H7-directed antibodies has been mapped^58^ and validated^57,59^ for H7N9-specific vaccines. On the other hand, it has become well established that also anti-neuraminidase responses can play a decisive role also in the framework of seasonal vaccines^60^. Accordingly, the protective capacity and the mechanism of N9-directed antibodies has already been described^61^. Therefore, the protection demonstrated by our N9-encoding platform-based vaccine that induces high neuraminidase-inhibiting antibody titers is well in accordance with such mechanisms. Surprisingly, we found also neuraminidase inhibiting activity in sera of mice vaccinated with MV_vac2_-H7(P) when tested via ELLA. This unexpected finding can be potentially explained by steric hindrance of antibodies binding the H7 at the apical receptor-binding domain or at the conserved stalk region, thus either cross-shielding the closely associated N9 tetramer from interaction with its substrate or pushing the neuraminidase tetramer away from the hemagglutinin trimer and the thereof bound sialic acids as demonstrated for monoclonal hemagglutinin-directed antibodies^62^.

One potential limitation of our study is the animal model used for the vaccine study. We have been using mice deficient in the type-I interferon receptor and transgenic for huCD46, IFNAR^−/−^-CD46Ge mice. Although these mice are defective for some aspects of innate immunity, they have been used as gold-standard small animal model for the analysis of MeV-derived recombinant vaccines due to lack of permissiveness of other fully immune competent animals for MeV replication besides non-human primates (NHPs). Nevertheless, a considerable number of studies have demonstrated transferability of immunogenicity and efficacy data from the IFNAR^−/−^-CD46Ge mice into NHPs or human patients, reviewed in^63^. Moreover, we have demonstrated for a MeV-derived MERS vaccine candidate that replication of the applied recombinant vaccine virus is required for the induction of immune responses in these animals, since UV-inactivated inoculum did not cause any immune reactions^64^. Few years ago after the start of this project it was demonstrated that the decisive factor for susceptibility of mice for in vivo replication of MeV is the IFNAR^−/−^ defect, while the CD46 transgene is having no obvious impact^65^. Therefore, this transgene may be abandoned in future in vivo studies.

In any case, our study provides proof-of-concept for analyzing the antigen format of highly pathogenic AIV hemagglutinin and neuraminidase in the backbone of a recombinant MeV in an otherwise naïve animal. Thus, we cannot predict on the basis of these data the impact of preformed measles immunity on the performance of our vectors. Interestingly, this impact seems to differ depending on the respective vaccine candidate. While MeV-derived vectors encoding antigens of HIV-1^22^ or CHIKV^23^ have been showing comparable induction of at least humoral responses in pre-immune and naïve mice, NHPs, or for MV-CHIKV even human patients^29,30^, the further development of a MeV-derived COVID-19 vaccine was stopped after early clinical trials revealed noncompetitive immunogenicity, which correlated negatively with the degree of pre-immunity^66^. Thus, parameters determining the impact of pre-immunity and the causes for the initially rather surprising perseverance of at least some MeV-derived vectors in the face of measles immunity remain to be understood. Nevertheless, MeV was chosen as the platform technology for providing proof of concept, here, also since recombinant MeV can be generated within less than a month. This is depicted by our studies concerning the MeV-derived COVID-19 vaccine, where complete immunogenicity data were available within 6 months after the SARS-CoV-2 sequence became available^67^ and animal experiments revealed to be the rate limiting step to prove efficacy^26^.

In summary, we were able to demonstrate with the recombinant measles vaccine technology, that both hemagglutinin and neuraminidase of the highly pathogenic IAV subtype H7N9 can be effective antigen targets for platform-based approaches. This adds another subtype to the list to preclinical studies proofing efficacy for MeV-derived vaccine candidates when encoding the hemagglutinin of seasonal influenza^68^, the 2009 pandemic H1N1 subtype^61,69^ or pre-pandemic highly pathogenic avian subtype H5N1^70^. Here, powerful cellular and humoral immune responses were induced against both H7 and N9 that were extremely long-lived in the case of H7-directed responses. Thereby vaccinated mice were completely (H7) or mostly (N9) protected against lethal challenge, validating side-by-side the efficacy of both antigens for the future design of platform-based pandemic influenza vaccines.

## Methods

### Cells

MDCK (*Canis familiaris* kidney) (ATCC CCL-34), Vero (African green monkey kidney) (ATCC CCL-81) and 293 T (ATCC CRL-3216) cell lines were purchased from ATCC (Manassas, VA, USA) and cultured in DMEM (Cat.No. BE12-741F, Lonza, Cologne, Germany) supplemented with 10% fetal bovine serum (FBS; Biochrom, Berlin, Germany) and 2 mM L-Gln (Biochrom). JAWSII dendritic cells (ATCC CRL-11904) were purchased from ATCC and cultured in MEM-α with ribonucleosides and deoxyribonucleosides (Cat.No. 22571020, GIBCO BRL, Eggenstein, Germany) supplemented with 20% FBS, 2 mM L-Gln, 1 mM sodium pyruvate (Biochrom), and 5 ng/ml murine GM-CSF (Peprotech, Hamburg, Germany). DC2.4 murine dendritic cells^71^ were cultured in RPMI 1640 (Cat.No. L0500-500, Biowest, Nuaillé, France) containing 10% FBS, 2 mM L-Gln, 1% non-essential aminoacids (Biochrom), 10 mM HEPES (pH 7.4), and 50 µM 2-mercaptoethanol (Sigma-Aldrich, Steinheim, Germany). All cells were cultured at 37 °C in a humidified atmosphere containing 6% CO_2_ for a maximum of 6 months of culture after thawing of the original stock.

### Plasmids

The HA and NA genes of A/Shanghai/2/2013 (H7N9) influenza virus were amplified by PCR flanked with *Aat*II*/Mlu*I binding sites using pCAGGS-H7 and pCAGGS-N9^72,73^ as templates and primer pairs H7 fwd / H7 rev and N9 fwd / N9 rev (Supplementary Tab. 1), respectively. Amplicons were cloned into pCR2.1-TOPO (Invitrogen Life technologies) according to manufacturer´s instructions, and fully sequenced. Both antigens, as well as the CMV promotor^74^, were inserted into p(+)BR-MV_vac2_-GFP(H) or p(+)MV_vac2_-ATU(P)^75^ via *Aat*II*/Mlu*I or *Sfi*I/*Sac*II, respectively, to generate p(+)PolII-MV_vac2_-H7(H), p(+)PolII-MV_vac2_-H7(P), p(+)PolII-MV_vac2_-N9(H), or p(+)PolII-MV_vac2_-N9(P). Thereby, the influenza virus antigens are encoded by measles genomes possessing otherwise Moraten and Schwarz vaccine strain-identical coding capacity. For construction of lentiviral transfervectors encoding H7, N9, or MeV nucleocapsid protein N, the respective ORFs were amplified by PCR with primers H7fwdNheI/H7revXhoI or N9fwdNheI/N9revXhoI (Supplementary Table 1), respectively, encompassing flanking *NheI/XhoI* restriction sites. PCR products were cloned into pCR2.1-TOPO (Invitrogen Life technologies) and fully sequenced. Intact antigen ORFs were cloned into pCSCW2gluc-IRES-GFP^76^ using *NheI/XhoI* restriction sites to yield pCSCW2-H7-IRES-GFP, pCSCW2-N9-IRES-GFP, or pCSCW2-MV-N-IRES-GFP, respectively.

### Production of lentiviral gene-transfer vectors

Lentiviral gene-transfer vectors were produced using 293 T cells and polyethylenimine (PEI) (Sigma-Aldrich) transfection^77^. In detail, 1×10^7^ 293 T cells were seeded per 175 cm^2^ cell culture flasks and cultured overnight. To produce VSV-G pseudotyped lentiviral vectors, these cells were transfected using a standard three-plasmid lentiviral vector system^78^. For transfection 17.5 μg pCSCW2-H7-IRES-GFP, pCSCW2-N9-IRES-GFP, or pCSCW2-MV-N-IRES-GFP transfer vector, 6.23 μg pMD2.G, and 11.27 μg pCMVΔR8.9^79^ in 1.5 ml DMEM w/o additives were mixed with 1.5 ml DMEM containing 18 mM polyethyleneimine by vortexing for 1 min. After 10 min incubation at RT, the transfection mixture was added dropwise into the T175 cell culture. The medium was exchanged the day after and [HIV_H7-IRES-GFP_(VSV-G)], [HIV_N9-IRES-GFP_(VSV-G)], or [HIV_MV-N-IRES-GFP_(VSV-G)] vector particles were harvested two and three days after transfection. For the harvest of vector particles, the supernatant of transfected culture flasks was filtered (0.45 μm), pooled, if applicable, and concentrated by ultracentrifugation (100,000 × g, 3 h, 4 °C). Supernatants were discarded, vector-containing pellets suspended in DMEM, aliquoted, and stored at −80 °C.

### Generation of antigen-expressing cell lines

Syngeneic antigen-presenting cells, i.e. the C57BL/6-derived DC lines JAWSII or DC2.4, were transduced with [HIV_H7-IRES-GFP_(VSV-G)] or [HIV_N9-IRES-GFP_(VSV-G)] vector-containing supernatant in dilution series to select singly transduced cell clones, which co-express H7 or N9 and GFP (JAWSII_green_-H7, JAWSII_green_-N9, DC2.4_green_-H7, and DC2.4_green_-N9, respectively). Syngeneic DC line JAWSII was transduced with [HIV_MV-N-IRES-GFP_(VSV-G)] to co-express MeV-N and GFP (JAWSII_green_-MV-N). For analysis of transduction efficiencies, cells were fixed in 1% paraformaldehyde (Merck Millipore, Darmstadt, Germany), and the percentage of GFP-positive cells was quantified by flow cytometry using an LSRII flow cytometer (BD, Heidelberg, Germany). Cell populations revealing a 1% to 10% fraction of GFP-positive cells were used for single-cell cloning by limiting dilution. For that purpose, dilutions of cell suspensions with 50 μl conditioned medium statistically containing 0.3 cells were seeded per well in 96-well plates. Single cells clones were cultured and analyzed by flow cytometry. GFP-positive clones were selected for further analysis. These transduced cell clones express present the respective processed antigen-epitopes of H7 or N9, or for control purpose MeV-N via MHC-I^24^.

### Viruses

Recombinant MeV were rescued according to the method of Martin et al. ^74^. In brief, 5 μg of MeV genome plasmids with H7- or N9-expression cassettes were co-transfected with pCA-MV-N (0.4 μg), pCA-MV-P (0.1 μg), and pCA-MV-L (0.4 μg) encoding MeV RNP proteins into 293 T cells cultured in 6-well plates using Lipofectamine 2000 (Invitrogen Life Technology) according to the manufacturer´s instructions. The transfected 293 T cells were overlaid two days after transfection onto 50% confluent Vero cells seeded in 10 cm-dishes the day before. Overlay cultures were closely monitored for isolated syncytia indicating monoclonal replicative centers. Single syncytia were picked and incubated with 50% confluent Vero cells cultured in 6-well plates. This “passage 0” (P0) virus clones were harvested by scraping all cells into the culture medium at the time of maximal infection. Cells were broken up by one freeze-thaw cycle, cellular debris removed by centrifugation (3000 × g, 5 min, 4 °C), the virus-containing supernatant was aliquoted and stored at -80 °C. Subsequent passages were generated after TCID_50_ titration of infectious virus by limiting dilution according to the method of Kaerber and Spaerman^80^ and infection of Vero cells at a multiplicity of infection (MOI) of 0.03. Selected single virus clones were passaged up to P10.

Multistep viral growth kinetics of recombinant MeV were analysed by infecting Vero cells at an MOI of 0.03 in 12-well plates and incubating the cultures at 37 °C. At various time points, supernatants were clarified by centrifugation, and cells were scraped into OptiMEM and subjected to freeze-thaw cycles. Released and cell-associated viral titers were determined by TCID_50_ titration of infectious virus by limiting dilution according to the method of Kaerber and Spaerman^80^.

Generation by reverse genetics of the 2:6 recombinant IAV containing hemagglutinin and neuraminidase of A/Shanghai/2/2013 (H7N9) and the other 6 gene segments of the laboratory strain A/Puerto Rico/8/1934 (H1N1) (A/Shanghai/2/2013-A/PR/8/34 (H7N9)) was described^73^. This recombinant virus was used for IPMA, neutralization and hemagglutination inhibition assay (HAI). The wild type zoonotic H7N9 virus isolate used for challenge experiments, A/Anhui/1/2013, was kindly provided by John McCauley, Crick Worldwide Influenza Centre, London, United Kingdom. Influenza viruses were propagated in MDCK cells (cells washed 2x with PBS before) in the presence of 1 µg/ml TPCK-treated trypsin (Sigma-Adrich) in the cell infection medium (DMEM, 2 mM L-Gln, 0.6% BSA) and were harvested in aliquots after 48 h at appearance of a clear cytophathic effect. The viral stocks were titrated using plaque assay under semi-solid overlay medium^81^. Briefly, 90% confluent MDCK cells grown in 6-well plates were incubated with 10-fold serial dilutions of the virus stock diluted in virus dilution medium (DMEM, 1 mM L-Gln, 0.1% BSA) for 1 h. Subsequently, overlay medium (1× EMEM (Lonza, Basel, Switzerland) supplemented with 1.5% Avicel-RC 591 (FMC BioPolymer), 2 mM L-Gln, 0.1% BSA, 0.75 μg/ml TPCK-trypsin) was added to infected cells after removal of infection medium. Cells were then fixed 2-3 days post infection with 4% formaldehyde solution after removal of overlay medium and plaques were subsequently counter-stained with 0.1% crystal violet solution (20% MeOH). All virus stocks were stored in aliquots at -80 °C.

### Sequence analysis of MeV genomes

The RNA genomes of recombinant MeV in P3 or P10 were isolated using the QIAamp RNeasy Kit (QIAgen, Hilden, Germany) according to manufacturers´ instructions, re-suspended in 50 μl RNase-free water and either directly used or stored at -20 °C. Viral cDNA was reversely transcribed using Superscript II RT kit (Invitrogen) with 2 μl vRNA as template and random hexamer primers, according to manufacturer´s instructions. For specific amplification of the genomic regions of interest, the respective stretches were amplified by PCR using primers binding to flanking sequences and cDNA as template. Detailed description of primers and procedures are available upon request. The gel-purified PCR products were subsequently sequenced (Eurofins Genomics, Ebersberg, Germany).

### Western Blot analysis

For Western Blot analysis, infected cells were lysed in lysis buffer (50 mM Tris pH 8.0; 62.5 mM EDTA; 1% NP-40; 0.4% deoxycholate; 40 μl/ml protease inhibitor cocktail (25×; Roche, Mannheim, Germany)). Cell lysates were denatured by incubation for 10 min at 95 °C in 2× urea sample buffer (5% SDS, 8 M urea, 200 mM Tris-HCl, 0.1 mM EDTA, 0.03% bromphenol blue, 2.5% DTT, pH 8.0), separated by electrophoresis on 10% SDS-PAGE gels and electrotansferred onto nitrocellulose membranes (GE Healthcare, Freiburg, Germany). The membranes were blocked with 1% milk powder in PBS and subsequently incubated with primary antibodies for 1 h at RT. A polyclonal rabbit serum raised against influenza virus A/FPV/Rostock/1934 (H7N1) (1:5000) was used as primary antibody to detect H7, and rabbit anti-MeV-N polyclonal antibody (1:25,000) (Cat.No. ab23974; Abcam, Cambridge) for MeV-N detection. Donkey HRP-coupled anti-rabbit IgG (H&L) serum (1:10,000) (Cat.No. 611-7302; Rockland, Gilbertsville, PA) served as secondary antibody in both staining procedures. Peroxidase activity was visualized with the enhanced chemiluminescence detection kit (Thermo Scientific, Bremen, Germany) on Amersham Hyperfilm ECL films (GE Healthcare).

### Neuraminidase activity assay

1 × 10^4^/well Vero cells cultured in 96-well black polystyrene plates with clear bottom (Corning, Wiesbaden, Germany) were infected at an MOI of 0.05 to 5 with MV_vac2_-N9(H) or MV_vac2_-N9(P) for 48 hours at 37 °C. Supernatants were removed, and 50 μL of 0.2 mM 2′-(4-Methylumbelliferyl)-α-D-N-acetylneuraminate (MUNANA) (Sigma, stock in N,N-dimethylformamide) diluted in NA-assay buffer (4 mM CaCl_2_ in TBS, pH 7.0) was added. Plates were incubated for 30 min at 37 °C. 100 μL NA-assay stop buffer (pH 10.7; 25% ethanol; 0.1 M glycine; 0.3% Tween 20; in H_2_O) was added and plates were incubated for 30 min at 37 °C. Free 4-methylumbelliferone was determined using a spectrofluorometer (excitation at 365 nm, emission at 450 nm). Concentration of 4-methylumbelliferone was calculated according to a 4-methylumbelliferone salt (Sigma) standard curve.

### Animal experiments

All animal experiments were carried out in compliance with the regulations of the German animal protection law and have been authorized by the RP Darmstadt. Six- to 12-week-old IFNAR^−/−^-CD46Ge mice^28^ were inoculated intraperitoneally (i.p.) with 1 × 10^5^ TCID_50_ of recombinant MeV or 200 μl OptiMEM on days 0 and 28, and bled via the retrobulbar route on days 0, 28, and 32 or 49 p.i. under anesthesia. Mice were euthanized on days 32 or 49 p.i., and spleens were isolated. Serum samples were stored at -20 °C. For challenge experiments, immunized mice were challenged i.n. 21 days post booster immunization with 10-fold LD_50_ dose (10^4^ pfu) of A/Anhui/1/2013 (H7N9) strain in 30 μl. Survival of infected mice were checked for maximal 21 days (cutoff criteria >20% weight loss) or until weight had normalized to the initial value.

For H7N9 neutralization and hemagglutination inhibition assay, sera were heat-inactivated for 30 min at 56 °C and treated with 20% RDE (receptor destroying enzyme) at 37 °C overnight. Treated sera were 1:2 diluted with 1.5% sodium citrate solution, incubated at 56 °C for 30 minutes, and stored at -20 °C.

### Immunoperoxidase monolayer assay (IPMA)

1 × 10^4^/well MDCK cells cultured in 96-well plates were infected at an MOI of 0.2 with recombinant influenza virus strain A/Shanghai/2/2013-A/PR/8/34 (H7N9). Two days post infection, infectious supernatants were discarded and the plates were heat-dried at 65 °C for 8 h. 50 μl of serially 2-fold diluted mice sera (starting from 1:8 dilution) were incubated with the dried cells for 2 h at room temperature. After 1x washing with PBS, the plates were incubated with peroxidase-conjungated rabbit-anti-mouse antiserum (1:750 in PBS) (Cat.No. P0260; H&L, Dako) for 1 h at room temperature. 3-amino-9-ethylcarbazole (AEC) substrate solution was prepared according to the manufacturer’s instructions using AEC dissolved in N,N-dimethylformamide (Merck Millipore) and used for visualization of infected cells. The reaction was stopped by addition of H_2_O. Antibody titers were calculated as the reverse of the highest sera dilution allowing staining of infected cells.

### Neutralization assays

For quantification of virus neutralizing titers (VNT), mouse sera were two-fold serially diluted in DMEM. For testing neutralization of MeV, 50 PFU of MV_vac2_-GFP(P)^64^ were mixed with diluted sera, and incubated at 37 °C for 1 h. The serum-treated virus suspensions were added to 1 × 10^4^ Vero cells seeded 4 h prior to assay in 96-well plates and incubated for 4 d at 37 °C. Vero cells were subsequently analyzed for syncytia formation, and VNT was calculated as reciprocal of the highest serum dilution that fully abrogated infectivity. For testing neutralization of H7N9 influenza virus, 200 TCID_50_ of H7N9 A/Shanghai/2/2013-A/PR/8/34 virus were incubated with two-fold serial sera dilutions for 20 min at room temperature. 2 × 10^4^ MDCK cells were added to the virus suspension in 96-wells and incubated at 37 °C for 2 d. Then, infected cells were immune-stained using a polyclonal ferret anti-PR/8 serum to visualize infection by non-neutralized influenza virus. Virus neutralizing titers (VNT) were calculated as the reciprocal of the highest dilution abolishing infection.

### Hemagglutination inhibition assay

4 hemagglutinating units (HAU) of A/Shanghai/2/2013-A/PR/8/34 (H7N9) in 25 µl PBS were added to 25 μl of 2-fold serially diluted sera in 96-U-well plates (Nunc) starting with an initial dilution of 1:10. Following incubation for 30–45 min at room temperature, 50 µl of a solution of 0.75% chicken red blood cells (RBCs) in Alsever’s solution (2.05% dextrose, 0.8% sodium citrate, 0.055% citric acid, and 0.42% sodium chloride) were added. The plates were incubated at room temperature until hemagglutination was observed. The HAI titer was calculated as the reciprocal of the highest serum dilution preventing hemagglutination of RBCs by influenza virions.

### Enzyme-linked lectin assay (ELLA)^38^

Flat 96-well plates with high protein-binding capacity (Nunc MaxiSorp) were coated overnight with 100 µl fetuin (25 μg/ml; Sigma-Aldrich) in 0.1 M PBS. To determine concentration of the probe to be used for ELLA, A/Shanghai/2/2013-A/PR/8/34 (H7N9) suspension was serially diluted in Dulbecco’s PBS with supplements (pH 7.4, 0.9 mM CaCl_2_, 0.5 mM MgCl_2_, 1 % BSA, 0.5 % Tween 20). 50 µl of sample diluent was added into the fetuin-coated plates. 50 µl of virus dilutions were afterwards dispensed into fetuin-coated plates. Plates were incubated 16-18 h at 37 °C. After washing 6× with PBS containing 0.05% Tween 20 (PBS-T), 100 μl peanut agglutininin conjugated to horse-radish peroxidase solution (PNA-HRPO, Sigma) were added per well and incubated for 2 h at RT. After 3× washing with PBS-T, 100 μl o-phenylenediamine dihydrochloride substrate (OPD, Sigma) were added per well and incubated for 10 min in the dark, before the reaction was stopped by addition of 100 μl sulfuric acid (1 N) per well. Finally, plates were read at 490 nm. The virus dilution resulting in 90-95 % of maximum signal was chosen as the appropriate concentration for testing the anti-neuraminidase activity of the respective mouse sera. Sera of mice were heat-inactivated at 56 °C for 30 min and 2-fold serially diluted in PBS starting with an initial dilution of 1:5. 50 μl of sera dilutions in duplicates were dispensed into fetuin-coated plates, and 50 μl of the previously determined virion dilution added. ELLA was performed as described above. The mean absorbance of the background (Abkg) was subtracted from the test wells and positive control (Apos) wells. The percent NA activity was calculated by dividing the mean absorbance of duplicate test wells (Atest) by the mean absorbance of virus-only wells and multiplied by 100, i.e. (Atest − Abkg)/(Apos − Abkg) × 100. To determine percent NA inhibition, the percent activity was subtracted from 100. The 50% end-point NAI titer was defined as the reciprocal of the highest dilution resulting in at least 50% inhibition of maximum signal in the assay.

### ELISpot assays

Murine IFN-γ ELISpot assays were purchased (ebioscience, Frankfurt, Germany) and performed according to manufacturer´s instructions using Multiscreen-IP ELISPOT PVDF 96-well plates (Millipore, Darmstadt, Germany). For this purpose, 5 × 10^5^ splenocytes isolated 4 d after booster immunization were co-cultured with 5 × 10^4^ JAWSII_green_- or DC2.4_green_-cells transgenic for H7 or N9, or the unmodified DC cell lines in 200 μl RPMI with 10% FBS, 2 mM L-Gln and 1 IU/ml Penicillin/ 100 µg/mL Streptomycin in above mentioned ELISPOT PVDF 96-well plates. Medium alone served as negative control. 10 μg/ml Concanavalin A (ConA, Sigma-Aldrich, St. Louis, MO) was used for general stimulation of splenocyte reactivity. 10 μg/ml MeV bulk antigens (Virion Serion, Würzburg, Germany) were used to stimulate MeV-specific T cells. After 36 h of stimulation, the splenocytes were removed from the plates, which were subsequently incubated with biotin-conjugated anti-IFN-γ antibodies and avidin-HRP according to the manufacturer´s instructions. 100 µl of 3-amino-9-ethyl-carbazole (AEC, Sigma-Aldrich) chromogen dissolved in N,N-dimethylformamide (Merck Millipore) was used for staining of spots. The reaction was stopped by washing with H_2_O. Spots were counted using an Eli.Scan ELISpot Scanner (A.EL.VIS, Hamburg, Germany) and ELISpot Analysis Software (A.EL.VIS).

### Statistical analysis

To compare the means of different groups in growth curves, neutralization assay, and ELISpot, non-parametric One-way ANOVA was applied.

### Material availability statement

Unique biological materials used in this study are available via commercial sources were indicated or via the corresponding author of this study upon reasonable request. (Recombinant) viruses or plasmids may require filing of materials transfer agreements (MTAs) and compensation for shipment.

### Reporting summary

Further information on research design is available in the Nature Research Reporting Summary linked to this article.

## Supplementary information

Supplemental Information

REPORTING SUMMARY

## Data Availability

All data generated or analysed during this study are included in this published article (and its supplementary information files).
