# Supplementary material for: A protective measles virus-derived vaccine inducing long-lasting immune responses against influenza A virus H7N9
Source: NPJ Vaccines. 2023 Mar 24;8:46. doi: 10.1038/s41541-023-00643-9 (PMC10037405; doi:10.1038/s41541-023-00643-9)
Supplement: Supplementary file 1 — Supplemental Information [file 41541_2023_643_MOESM1_ESM.pdf]

Supplementary Tab. 1: Primers

**Name**	**Sequence (5‘ – 3‘)**
H7 fwd	ACG CGT ATG AAC ACT CAA ATC CTG G
H7 rev	GAC GTC TCA TTA TAT ACA AAT AGT GCA CCG
N9 fwd	ACG CGT ATG AAT CCA AAT CAG AAG ATT C
N9 rev	GAC GTC TTA GAG GAA GTA CTC TAT TTT AG
H7fwdNheI	CTT GCT AGC ATG AAC ACT CAA ATC CTG GTA TTC GCT CTG
H7revXhoI	CTT CTC GAG TCA TTA TAT ACA AAT AGT GCA CCG CAT GAA TC
N9fwdNheI	CTT GCT AGC ATG AAT CCA AAT CAG AAG ATT CTA TGC ACT TC
N9revXhoI	CTT CTC GAG TTA GAG GAA GTA CTC TAT TTT AGC CCC ATC

a

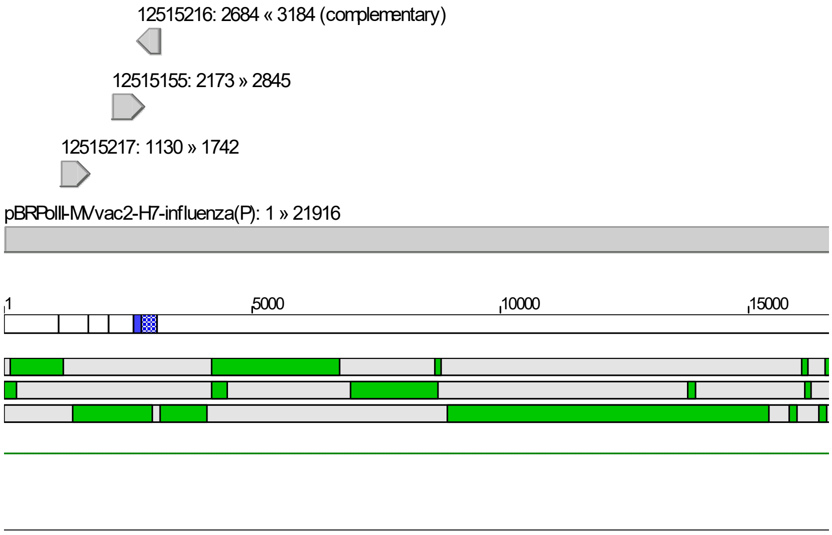

b

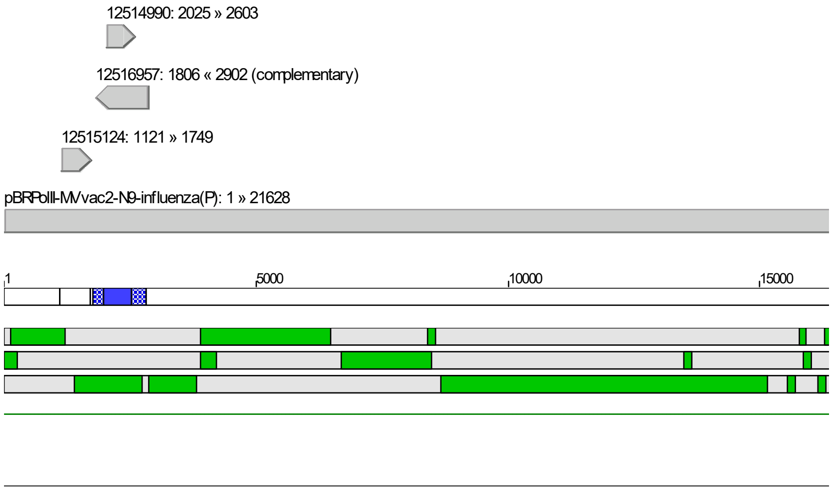

Supplementary Fig. 1: Alignment of antigen-encoding sequences of P3 vaccine viruses. Alignment of sequence reads for antigen-encoding additional gene cassettes of (**a**) MV_{vac2}-H7(P) and (**b**) MVvac2-N9(P) in passage 3 with expected vector genome sequences. Absence of divergences in the alignment demonstrates identity of the genes and absence of mutations during cloning, rescue, and passaging of recombinant vaccine viruses.

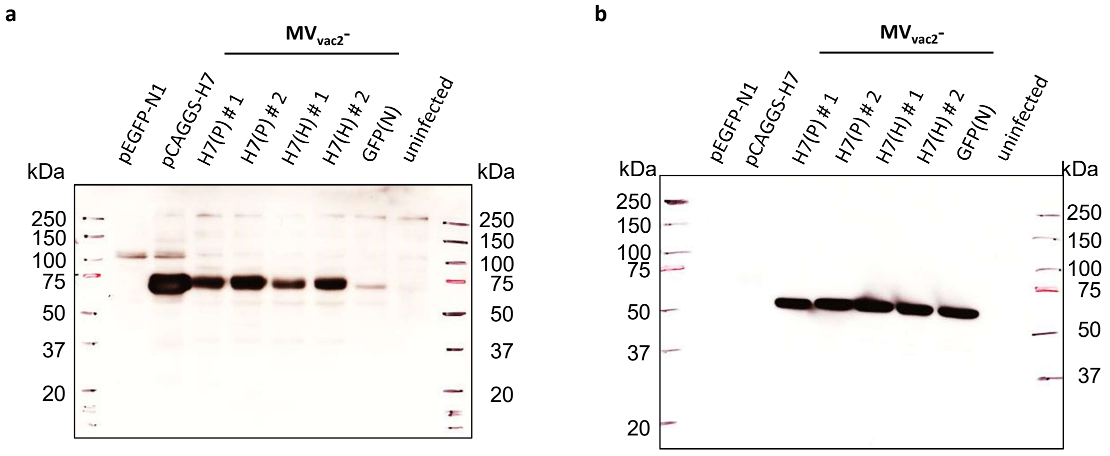

Supplementary Fig. 2. Expression of influenza virus H7 in Vero cells infected with MV$_{vac2}$-H7(P) and MV$_{vac2}$-H7(H). Non-cropped blots of Western blot analyses presented in Fig. 1c. Immunoblot analysis of Vero cells infected at an MOI of 0.03 with two independent virus clones of MV$_{vac2}$-H7(H) and MV$_{vac2}$-H7(P) or MV$_{vac2}$-GFP(N) as depicted. Membranes were probed using (a) rabbit serum reactive against H7N1 or (b) mAb reactive against MeV-N. H7 expression plasmid pCAGGS-H7 transfected into 293T cells served as positive control for H7 expression, EGFP expression plasmid pEGFP-N1 as negative control for transfected 293T cells.

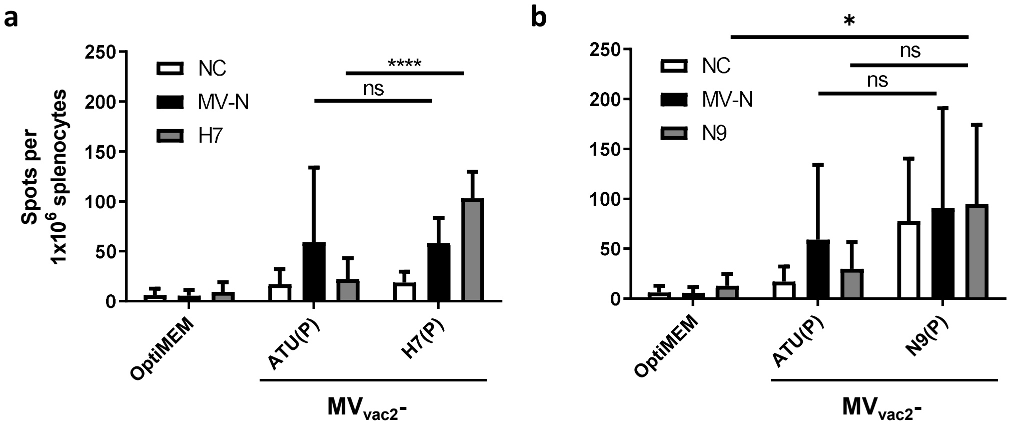

Supplementary Fig. 3: Secretion of IFN-γ after antigen-specific re-stimulation of splenocytes with DC2.4 dendritic cell lines. IFN-γ ELISpot analysis of murine splenocytes isolated 4 d after boost immunization. (**a**) H7- or (**b**) N9-specific T cells were detected after co-culture of splenocytes with DC2.4 dendritic cell lines transgenic for MeV-N (black columns), H7 or N9 (grey colums). (**a, b**) Untransduced cells (NC) served as negative controls (white columns). Respective positive and negative stimulation controls are shown in Fig. 4c. Presented are means and standard deviations per group (n = 6). ns, not significant; *, $p < 0.05$; ****, $p < 0.0001$.
