# Supplementary material for: A protective measles virus-derived vaccine inducing long-lasting immune responses against influenza A virus H7N9
Source: NPJ Vaccines. 2023 Mar 24;8:46. doi: 10.1038/s41541-023-00643-9 (PMC10037405; doi:10.1038/s41541-023-00643-9)
Supplement: Supplementary file 2 — REPORTING SUMMARY [file 41541_2023_643_MOESM2_ESM.pdf]

nature portfolio

Corresponding author(s): Michael D. Mühlebach

Last updated by author(s): Jan 30, 2023



# Reporting Summary

Nature Portfolio wishes to improve the reproducibility of the work that we publish. This form provides structure for consistency and transparency in reporting. For further information on Nature Portfolio policies, see our Editorial Policies and the Editorial Policy Checklist.

## Statistics

For all statistical analyses, confirm that the following items are present in the figure legend, table legend, main text, or Methods section.

| n/a | Confirmed | |
|---|---|---|
| ☐ | ☒ | The exact sample size (*n*) for each experimental group/condition, given as a discrete number and unit of measurement |
| ☐ | ☒ | A statement on whether measurements were taken from distinct samples or whether the same sample was measured repeatedly |
| ☐ | ☒ | The statistical test(s) used AND whether they are one- or two-sided<br>*Only common tests should be described solely by name; describe more complex techniques in the Methods section.* |
| ☒ | ☐ | A description of all covariates tested |
| ☒ | ☐ | A description of any assumptions or corrections, such as tests of normality and adjustment for multiple comparisons |
| ☐ | ☒ | A full description of the statistical parameters including central tendency (e.g. means) or other basic estimates (e.g. regression coefficient) AND variation (e.g. standard deviation) or associated estimates of uncertainty (e.g. confidence intervals) |
| ☒ | ☐ | For null hypothesis testing, the test statistic (e.g. *F*, *t*, *r*) with confidence intervals, effect sizes, degrees of freedom and *P* value noted<br>*Give P values as exact values whenever suitable.* |
| ☒ | ☐ | For Bayesian analysis, information on the choice of priors and Markov chain Monte Carlo settings |
| ☒ | ☐ | For hierarchical and complex designs, identification of the appropriate level for tests and full reporting of outcomes |
| ☒ | ☐ | Estimates of effect sizes (e.g. Cohen's *d*, Pearson's *r*), indicating how they were calculated |

*Our web collection on statistics for biologists contains articles on many of the points above.*

## Software and code

Policy information about availability of computer code

| | |
|---|---|
| Data collection | n/a |
| Data analysis | Statistical analysis of data sets was performed using commercially available software: GraphPad Prism 9.2.0 |

For manuscripts utilizing custom algorithms or software that are central to the research but not yet described in published literature, software must be made available to editors and reviewers. We strongly encourage code deposition in a community repository (e.g. GitHub). See the Nature Portfolio guidelines for submitting code & software for further information.

## Data

Policy information about availability of data

All manuscripts must include a data availability statement. This statement should provide the following information, where applicable:

- Accession codes, unique identifiers, or web links for publicly available datasets
- A description of any restrictions on data availability
- For clinical datasets or third party data, please ensure that the statement adheres to our policy

All data generated or analysed during this study are included in this published article (and its supplementary information files).

## Human research participants

Policy information about studies involving human research participants and Sex and Gender in Research.

| | |
|---|---|
| Reporting on sex and gender | n/a |
| Population characteristics | n/a |
| Recruitment | n/a |
| Ethics oversight | n/a |

Note that full information on the approval of the study protocol must also be provided in the manuscript.

# Field-specific reporting

Please select the one below that is the best fit for your research. If you are not sure, read the appropriate sections before making your selection.

☒ Life sciences ☐ Behavioural & social sciences ☐ Ecological, evolutionary & environmental sciences

For a reference copy of the document with all sections, see nature.com/documents/nr-reporting-summary-flat.pdf

# Life sciences study design

All studies must disclose on these points even when the disclosure is negative.

| | |
|---|---|
| Sample size | Sample size n = 6 was calculated for a power of 80% assuming an effect size of 0.8 (standard deviation of the group means in correlation to the standard deviations within the groups) to detect a significance of $p < 0.05$. |
| Data exclusions | No data were excluded from this study |
| Replication | In vitro experiments were repeated twice, in general to ensure reproducibility. Animal experiments were usually performed in at least two batches each including all cohorts (due to animal availability) and results were pooled |
| Randomization | Animals were randomized according to sex and age to ensure eqwual distribution across experimental cohorts |
| Blinding | Blinding was not relevant in animal studies due to objective read-outs |

# Reporting for specific materials, systems and methods

We require information from authors about some types of materials, experimental systems and methods used in many studies. Here, indicate whether each material, system or method listed is relevant to your study. If you are not sure if a list item applies to your research, read the appropriate section before selecting a response.

### Materials & experimental systems

| n/a | Involved in the study | |
|---|---|---|
| ☐ | ☒ | Antibodies |
| ☐ | ☒ | Eukaryotic cell lines |
| ☒ | ☐ | Palaeontology and archaeology |
| ☐ | ☒ | Animals and other organisms |
| ☒ | ☐ | Clinical data |
| ☒ | ☐ | Dual use research of concern |

### Methods

| n/a | Involved in the study | |
|---|---|---|
| ☒ | ☐ | ChIP-seq |
| ☒ | ☐ | Flow cytometry |
| ☒ | ☐ | MRI-based neuroimaging |

## Antibodies

| | |
|---|---|
| Antibodies used | 1.) Polyclonal rabbit serum raised against influenza virus A/FPV/Rostock/1934 (H7N1)<br>2.) rabbit anti-MeV-N polyclonal antibody, Abcam, Cat.No. ab23974<br>3.) Donkey HRP-coupled anti-rabbit IgG (H&L) serum, Rockland, Gilbertsvile, PA, Cat.No. 611-7302<br>4.) peroxidase-conjungated rabbit-anti-mouse antiserum (H&L), Dako, Cat.No. P0260 |
| Validation | ad 1.) internal validation in experiments - inclusion of lysates of cells infected by vector w/o antigen |

| | |
|---|---|
| Validation | ad 2.) see manufacturer´s website: https://www.abcam.com/measles-nucleoprotein-antibody-ab23974.html<br>ad 3.) see manufacturer´s website: https://www.rockland.com/categories/secondary-antibodies/rabbit-igg-hl-antibody-peroxidase-conjugated-611-7302/<br>ad 4.) see manufacturer´s website: https://www.agilent.com/en/library/eifu?partNumber=P026002-2 |

## Eukaryotic cell lines

Policy information about cell lines and Sex and Gender in Research

| | |
|---|---|
| Cell line source(s) | Cell lines were purchased from ATCC (Manassas, VA, USA) |
| Authentication | Cells were purchased and cultured for no longer than 6 months after thawing of the original stock |
| Mycoplasma contamination | All cell lines were tested for absence of mycoplasma at least every 3 months, and no contamination has been detected |
| Commonly misidentified lines (See ICLAC register) | No commonly misidentified lines were used in this study |

## Animals and other research organisms

Policy information about studies involving animals; ARRIVE guidelines recommended for reporting animal research, and Sex and Gender in Research

| | |
|---|---|
| Laboratory animals | 6- to 12-week-old IFNARko-CD46Ge mice were used in this study |
| Wild animals | This study did not involve wild animals |
| Reporting on sex | Both sexes were used in this study. No obviious differences were found, but experiments in the study were not powered to detect minor differences due to sex. |
| Field-collected samples | The study did not involve samples collected from the field |
| Ethics oversight | All animal experiments were carried out in compliance with the regulations of the German animal protection law and have been authorized by the RP Darmstadt |

Note that full information on the approval of the study protocol must also be provided in the manuscript.
